# β-cell senescence in type 2 diabetes

**DOI:** 10.18632/aging.102502

**Published:** 2019-11-25

**Authors:** Cristina Aguayo-Mazzucato, Ayush Midha

**Affiliations:** 1 Joslin Diabetes Center, Harvard Medical School, Boston, MA 02215, USA

**Keywords:** beta-cells, senescence, senolysis, type 2 diabetes, insulin resistance

Age is one of the major risk factors for the development of type 2 diabetes mellitus (T2D). However, the understanding of how cellular aging contributes to diabetes pathogenesis is incomplete and as a result, current therapies do not target this aspect of the disease. Pancreatic β-cells play a central role in the development of T2D; healthy β-cells compensate for insulin resistance, and β-cell dysfunction causes the progression to overt diabetes. Normal β-cell compensatory mechanisms include an increase in mass through cellular proliferation and increased function, which manifests as hyperinsulinemia to maintain normal blood glucose levels.

Over time, β-cell compensation for insulin resistance may fail, resulting in a progressive decline of insulin secretion [1]. As a consequence, subjects progress from normal glucose tolerance (NGT) to impaired glucose tolerance (IGT) and, finally, to established T2D. Even after diagnosis, β-cell function continues to worsen. Early interventions to save β-cell function are a promising strategy to halt the progression of diabetes.

In recent work [2,3] we showed that insulin resistance induced the expression of aging markers, suggesting that β-cell aging could accelerate the progression toward diabetes. Therefore, reversing the hallmarks of cellular aging presents a potential avenue for novel T2D therapies; in particular, transcriptomic analysis of aged β-cells pointed us toward cellular senescence as a promising target. Senescent cells enter a state of long-term growth inhibition and replicative arrest after exposure to environmental insults, including genomic damage, oncogene activation, and reactive oxygen species [4]. The resulting changes in gene expression impair cell function and proliferation while modifying intercellular signaling through the senescence-associated secretory phenotype (SASP) [5]. The potential paracrine effects of senescent β-cells highlight the importance of the β-cell SASP in driving metabolic dysfunction.

Along these lines, we demonstrated that senescent β-cells downregulated hallmark identity genes, upregulated disallowed genes, and secreted proinflammatory cytokines [2]. We established two models of insulin resistance in mice: one using the delivery of the insulin receptor antagonist S961, and the other using a more physiologically representative high fat diet. In both cases, the metabolic stress increased the number of senescent β-cells while impairing glucose tolerance. Aging and SASP genes were also upregulated, but after insulin resistance was stopped, gene expression returned to healthy levels. This suggests that there might be critical windows during which β-cell senescence may be reversible. These results were consistent with experiments on human β-cells, in which senescence increased with age, body mass index and in the presence of T2D.

Additionally, we found that the targeted deletion of senescent cells, or senolysis, in mice improved β-cell function, reduced blood glucose levels, and restored healthy expression levels of aging and SASP genes. Our findings highlight the transformative therapeutic potential of senolytic drugs in restoring β-cell function among T2D patients.

The partial reversibility of β-cell senescence suggests that, consistent with recent publications [6], this is a non-binary phenomenon. External insults may create subpopulations of aged β-cells activating distinct levels of the senescence-associated regulatory progression.

The progression of damaged β-cells through this regulatory cascade likely accelerates T2D; eventually, the accumulation of senescent β-cells may cross a threshold inducing long-term metabolic dysfunction through the permanent loss of β-cell mass and function. The deletion of senescent β-cells or the reversal of senescence in a targeted subpopulation of aged β-cells may inhibit this cascade of dysfunction. To advance these therapeutic strategies, it is imperative to characterize the distinct subpopulations of senescent β-cells and the temporal expression patterns of senescence genes.

Such experiments will also elucidate how senolytic treatment affects β-cell mass. Given the central role of declining β-cell mass in T2D, recovering the cellular population’s proliferative capacity would permit compensatory increases in insulin production to lower blood glucose levels. Senolysis induces a short-term decrease in total β-cell number by actively deleting the senescent population. However, the treatment’s effects on β-cell function and glucose tolerance indicate that in the long-run, senolysis could restore the proliferative capacity of the remaining β-cells, both by avoiding glucotoxicity and by decreasing local SASP.

Inflammatory proteins specifically disrupt β-cell function by impairing glucose homeostasis, diminishing insulin secretion, and downregulating β-cell-specific transcription factors [7]. As a result, SASP factors may explain why the accumulation of senescent β-cells disturbs glucose tolerance and β-cell function. The secretory phenotypes of various subpopulations of senescent β-cells need to be studied to identify the specific mechanisms underlying aging-related loss of β-cell mass and function. Moreover, characterizing the β-cell SASP and the effects of its constituent factors will reveal further therapeutic targets for T2D.

These insights highlight a promising path forward for understanding the complex role of β-cell aging in T2D, but effective therapeutic translation requires further experimentation in human β-cells. Cultured human islets offer a useful platform for identifying potential drivers of senescence, characterizing the progression of β-cell aging, and validating candidate SASP factors. Furthermore, existing proof-of-concept clinical trials with senolytic drugs present a valuable opportunity to examine the efficacy and safety of senolytic therapy in reversing aging-related dysfunction in other tissues [8], some of which play key roles in glucose homeostasis. Continued investigation of β-cell senescence in animal models and human islets will enable similar trials to advance effective options for T2D therapy.
